# Dislocation and Fracture of Femur

**Published:** 1871

**Authors:** 


### *Dislocation and Fracture of Femur.*

In the Indiana Journal of Medicine, Dr. J. R. Weist of Richmond, Indiana, reports a case of dislocation of the head of femur upon the dorsum of the ilium, in a lady fifty-seven years of age, in which he, with Dr. Kersy, attempted the reduction six months after the injury. In the attempt at reduction by manipulation, and after the plan that Prof. Bigelow terms the method by "Rotation," the neck of the femur was fractured near the shaft. The limb was easily brought down upon a line with its fellow, and when left to itself was only shortened from a half to three-quarters of an inch. The patient was placed in bed and tension made by means of adhesive strips, weights and pulleys, for six weeks. At the end of this time union seeming sufficiently firm, all dressings were removed and the patient directed to make daily motion of the injured joint. After the patient commenced walking, the shortening increased from half to an inch and a quarter. As will be seen from the following extract from Dr. Weist's paper, the result was all that could have been reasonably hoped for under the circumstances:

When last seen, a little more than a year after the bone was fractured, Mrs. J—— could walk quite well without the aid of either crutch or cane. There was something of a limp in her gait, but this was not marked; a high-heeled shoe compensating for the deficient length of the limb. The injured hip remained weaker than the other, yet she reported herself able to keep up rather an active life in comparative comfort.

Nearly all of the natural motions of the limb could be executed to a fair degree, external rotation and abduction being most restricted. There was very little deformity about the hip. The head of the femur remained in about the position it was left after the failure at reduction.

